# Prolonged baleen hormone cycles suggest atypical reproductive endocrinology of female bowhead whales

**DOI:** 10.1098/rsos.230365

**Published:** 2023-07-26

**Authors:** N. S. J. Lysiak, S. H. Ferguson, C. A. Hornby, M. P. Heide-Jørgensen, C. J. D. Matthews

**Affiliations:** ^1^ Biology Department, Suffolk University, Boston, 02108, MA, USA; ^2^ Arctic Aquatic Research Division, Fisheries and Oceans Canada, 501 University Crescent, Winnipeg, Manitoba, Canada R3T 2N6; ^3^ Greenland Institute of Natural Resources, Nuuk, Greenland

**Keywords:** baleen, bowhead whale, calving interval, endocrine, gestation, progesterone

## Abstract

Serial measurements of hormone concentrations along baleen plates allow for reconstructions of mysticete whale reproductive histories. We assessed gestation and calving interval in bowhead whales (*Balaena mysticetus*) by measuring progesterone, oestradiol, corticosterone and nitrogen stable isotope ratios (δ^15^N) along baleen of 10 females from the eastern Canada-west Greenland population. Three immature females (body size < 14.32 m) had uniformly low progesterone concentrations across their baleen, while seven mature females (body size ≥ 14.35 m) had repeated, sustained elevations of progesterone indicative of pregnancies. The mean duration of progesterone elevations (23.6 ± 1.50 months) was considerably longer than the approximately 14 month gestation previously estimated for this species. We consider several possible explanations for this observation, including delayed implantation or sequential ovulations prior to gestation, strategies that would allow females to maximize their fitness in variable Arctic conditions, as well as suggest modified criteria defining gestation as a shorter component of the entire progesterone peak. Calving intervals varied within and among individuals (mean = 3.7 years; range = range 2.8–5.7 years), providing population-specific reproductive estimates for growth models used in bowhead whale management and conservation.

## 1. Introduction

Life-history traits such as age of maturation and reproductive rates are difficult to measure in large, free-ranging animals like cetaceans [1]. Reproductive knowledge of most populations comes from gross anatomical and physiological investigations of specimens collected from commercial or indigenous whaling [2–4]. Recent studies have used endocrine markers of various tissues to study health, stress and reproductive rates of baleen whales ([1,5–10], reviewed in [11]). Endocrine systems regulate body processes via negative feedback loops involving steroid hormones, which ultimately control reproductive processes such as maturation, oestrous and ovulation, pregnancy and seasonal mating behaviour [12]. For example, the female steroid hormone progesterone is produced at high concentrations during pregnancy and is a known proxy for gestation [13,14]. Glucocorticoids (GCs), such as cortisol and corticosterone, are biochemical markers of the vertebrate stress response, indicating activation of the hypothalamic-pituitary-adrenal axis in response to internal or external stressors [15,16].

The filter feeding baleen plates of mysticete whales grow continuously, and can be sampled longitudinally to construct retrospective chemical profiles to infer movement and foraging histories (via stable isotope analysis; e.g. [17,18]) or individual life history (via endocrine analysis; e.g. [19–21]). Validation studies have confirmed that progesterone elevations in baleen of female north Atlantic right whales (*Eubalaena glacialis*) correspond with known pregnancies [20,21]. Furthermore, baleen GCs (cortisol and corticosterone) and thyroid hormone concentrations were elevated during both internal (i.e. pregnancy) and external (e.g. fishing gear entanglement) stressors [20–24]. Baleen of the closely related bowhead whale (*Balaena mysticetus*) can exceed 4 m in length and hold upwards of 15–20 years of tissue growth [25], allowing for retrospective reconstructions of reproductive events over biologically meaningful time frames not possible with point measurements of tissues like skin or blubber.

Bowheads are the only Arctic-endemic baleen whale, and have evolved extreme longevity (exceeding 200 years, [26]), delayed sexual maturity (estimated at 25.8 years; [27]), and low reproductive rates [28,29], traits that probably maximize fitness in their Arctic environment. The eastern Canada-west Greenland (EC-WG) bowhead population is harvested seasonally in traditional Inuit hunts, with relatively low numbers (less than 3–5) taken annually. Although current hunts are well below levels that would induce population decline [30], EC-WG bowheads may be vulnerable to climate change-induced shifts in sea ice parameters that impact bottom-up [31,32] and top-down [33,34] drivers of population growth, and exacerbate anthropogenic threats such as shipping [35–37].

Population growth models require population-specific reproductive rates that are unknown for EC-WG bowhead whales, and surrogate parameters estimated for other populations (e.g. [38]) may differ owing to various factors (e.g. population structure and abundance, prey availability and carrying capacity, and levels of anthropogenic disturbance). Therefore, our study objectives were to assess gestation and associated stress responses, calving interval, and reproductive rate through sequential measurement of steroid hormones (progesterone, oestradiol and corticosterone) along baleen plates of 10 female EC-WG bowhead whales. In addition to informing reproductive parameters used in population modelling, this study provides new physiological data which, when married with existing field and gross observations, provide valuable and, until now, elusive, insights to the reproductive endocrinology of the bowhead whale.

## 2. Material and methods

### 2.1. Baleen specimens

**Figure 1.**
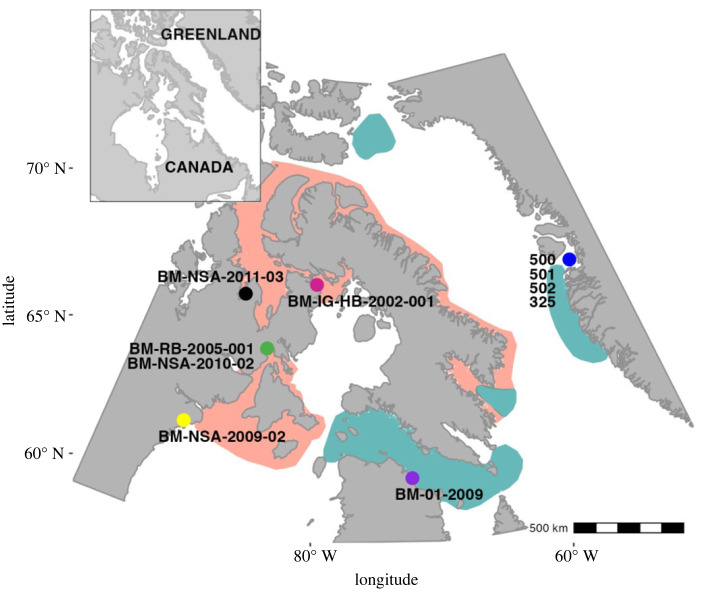
Bowhead whale hunt locations. Shading indicates approximate bowhead whale seasonal distribution in summer (red) or winter (green).

Baleen was collected from female EC-WG bowhead whales (*n* = 10) hunted by Inuit throughout the eastern Canadian Arctic and west Greenland from 1998–2011. In Canada, six whales were hunted during July to September from Hudson Bay, Hudson Strait, Foxe Basin, Gulf of Boothia, and northern and eastern Baffin Island [18], and four whales were hunted in Disko Bay, Greenland in April and May [39] (table 1; figure 1). Entire baleen plates including the most recent growth were excised from within the gum from three whales, while only erupted baleen cut at the gumline was collected from the other seven whales (table 1). Baleen was collected within 24–48 h of death, and frozen at –25°C. Total body length was measured for all whales and, when possible, eye lens and reproductive tract samples were collected during necropsy to assess whale age and reproductive status (table 1) using procedures detailed in Heide-Jørgensen *et al*. [40].

**Table 1.** Bowhead whale metadata, including hunt location and date, morphology, age, and gross examination of reproductive tracts, ranked by body size per sampling country. NU, Nunavut; QC, Quebec; GL, Greenland.

whale identity	hunt location	date	total length (m)	AAR age (yr)^a^	baleen length (cm)^a^	corpus lutei detected (Y/N)	fetus detected (Y/N)
	**EASTERN CANADIAN ARCTIC**						
NSA-2011-03	Gulf of Boothia, Kugaaruk, NU	08/2011	9.04	nd	176*	nd	nd
IG-HB-2002-001	Foxe Basin, Iglooik, NU	08/2002	14.19	32.0	263+	nd	nd
NSA-2010-02	Foxe Basin, Repulse Bay, NU	08/2010	14.32	nd	296*	nd	nd
NSA-2009-02	Hudson Strait, Rankin Inlet, NU	08/2009	16.15	45.1	232+	nd	nd
RB-2005-001	Foxe Basin, Repulse Bay, NU	08/2005	16.40	64.0	267+	nd	nd
01-2009	Hudson Strait, Kangiqsujuak, QC	08/2009	17.29	139.3	338*	nd	nd
	**GREENLAND**						
500	Disko Bay, GL	04/2010	14.35	36.4	236+	N	N
325	Disko Bay, GL	05/2009	15.50	41.9	192+	nd	nd
501	Disko Bay, GL	04/2010	15.85	40.8	262+	Y	N
502	Disko Bay, GL	05/2010	16.10	32.7	284+	Y	N

^a^AAR: age estimate via aspartic acid racemization of the eye lens; *total length (embedded+erupted baleen); +erupted length; nd: not determined.

### 2.2. Baleen sampling

Each baleen plate was cleaned using water and scrubbing pads, then scraped using a scalpel blade to remove adhered surface material. Starting at the proximal end, baleen samples were drilled every 2 cm along the outside edge using a hand-held rotary tool fitted with a 1/16-inch drill bit. Samples were collected until the tapered (distal) end of the baleen became too thin to collect the required 100 mg for hormone analysis, which typically excluded the final 10–20 cm of each plate. The 2 cm sample increments represent sub-seasonal, near monthly temporal resolution given annual baleen growth rates of 14–25 cm yr^−1^ in bowhead whales [17,18].

### 2.3. Hormone extraction

Baleen hormone extraction followed procedures developed by Hunt *et al*. [19,20,22,41], with some modifications. Six millilitres of 100% high-performance lipid chromatography-grade methanol was added to 100 mg of each baleen sample in 16 × 100 mm borosilicate glass tubes, which were capped and vortexed for 2 h, and then centrifuged for 15 min at 3000*g*. Then, 3.75 ml of the methanol supernatant containing hormones was transferred to a second borosilicate tube, dried down under an N_2_ stream for approximately 6 h, and frozen at –80°C. Dried hormone extracts were reconstituted in 1000 µl of enzyme immunoassay buffer (catalogue ‘X065’, Arbor Assays, Ann Arbor, MI, USA), vortexed, and pipetted to a cryovial for storage at –80°C prior to hormone assay. This solution is herein termed the ‘1:1’ (full-strength, neat) extract.

### 2.4. Hormone assays

Commercial enzyme immunoassay kits from Arbor Assays were used to analyse baleen progesterone (catalogue no. K025), corticosterone (catalogue no. K014) and 17β-oestradiol (catalogue no. K030). Progesterone and corticosterone were measured in all samples (i.e. at 2 cm increments), while oestradiol was measured at lower resolution (every 4 to 6 cm) that was increased to every 2 cm during some periods of elevated progesterone. An extensive laboratory validation study by Hunt *et al*. [41] demonstrated that all three assay antibodies exhibited reliable binding affinity to the desired hormone in bowhead whale baleen (i.e. good parallelism), and verified that each assay was able to distinguish a range of concentrations with acceptable accuracy.

The manufacturer's protocols were followed for analysis of all three hormones. Samples, standards, non-specific binding, and blank wells were assayed in duplicate. Any samples that fell outside 10–90% bound on the standard curve were re-assayed; samples with high hormone concentrations (per cent bound < 10%) were diluted 2-fold (1 : 2), or up to 1 : 128 in some high progesterone samples, while samples with low hormone concentrations (per cent bound > 90%) were concentrated within the assay wells at 2 : 1. Samples with more than 10% coefficient of variance between duplicates were re-assayed. Results were converted to nanograms of immunoreactive hormone per gram (ng g^−1^) of baleen. Baseline hormone concentrations were determined for each dataset using an iterative process excluding all points that deviate from the mean by 2 s.d. until no points exceed this threshold (after [42]).

### 2.5. Stable isotope analysis

Previous stable nitrogen isotope (*δ*^15^N) measurements along the baleen of the six whales hunted in Canada indicated annual cycling that most likely reflected foraging in isotopically distinct summering and wintering areas of their annual range [18]. We used these *δ*^15^N cycles to estimate annual baleen growth rates (see below) to interpret variation in hormone concentrations within a temporal context. *δ*^15^N of the four additional whales hunted in Greenland were measured at the same laboratory following the same protocols as the other six animals (details in [18]).

### 2.6. Data analysis

Average annual baleen growth rate across the entire baleen plate of each whale was estimated from spectra of autoregressive (AR) models [43,44] fitted to annual *δ*^15^N cycles (see [18]). Period (i.e. annual baleen growth rate) was estimated by converting the spectral peak frequency of each modelled hormone profile to samples per period (1/peak frequency) and then multiplying by the 2 cm sample increment.

**Figure 2.**
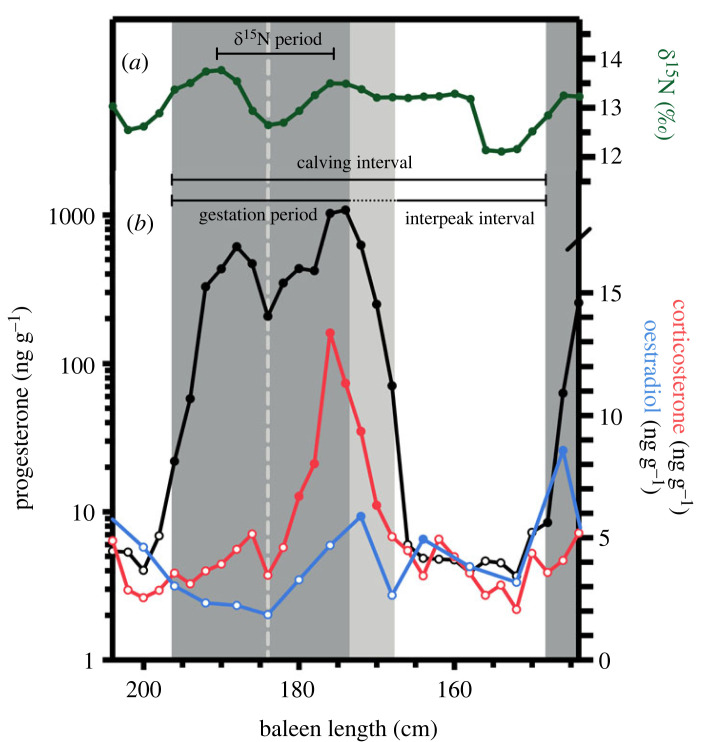
(*a*) Patterns in stable nitrogen ratios (*δ*^15^N, green) and (*b*) concentrations of immunoreactive progesterone (black), corticosterone (red) and oestradiol (blue) across a representative section of bowhead whale baleen (from individual 500). Closed circles indicate hormone concentrations above a baseline threshold, while open circles indicate hormone concentrations within baseline levels. Dark grey box highlights gestation as defined under hypothesis 2; light grey box extends gestation as defined under hypothesis 1. Light grey vertical dashed line demarcates first and second phase of P4 peak.

We tested two hypotheses for interpreting progesterone (P4) peaks, areas of elevated P4 concentration typically indicative of pregnancy status. Under *hypothesis 1*, we defined gestation as regions of baleen where P4 concentrations became elevated 2 standard deviations above baseline, remained so for more then 2 consecutive samples, and then returned to values at or below baseline (figure 2). The first and second phases (i.e. minima to minima) of the bimodal P4 peaks were also identified (see vertical dashed line, figure 2). Under *hypothesis 2*, peaks were defined similarly, but gestation ended when the final P4 maxima was reached—such that points on the decline back to P4 baseline values were excluded from calculations of gestation (figure 2). Only complete P4 peaks were included in subsequent analyses of gestation and calving interval. To estimate gestation, the length of baleen tissue containing each P4 peak was divided by the annual baleen growth rate (*δ*^15^N period). Interpeak interval was defined as a region of baleen following a P4 peak until the initiation of a subsequent peak (figure 2). To estimate the duration of each interpeak interval, the length of baleen separating P4 peaks was divided by the annual baleen growth rate, or *δ*^15^N period. Calving interval, or the time from one calving event to the next, was defined as gestation + the subsequent interpeak interval (figure 2).

## 3. Results

**Figure 3.**
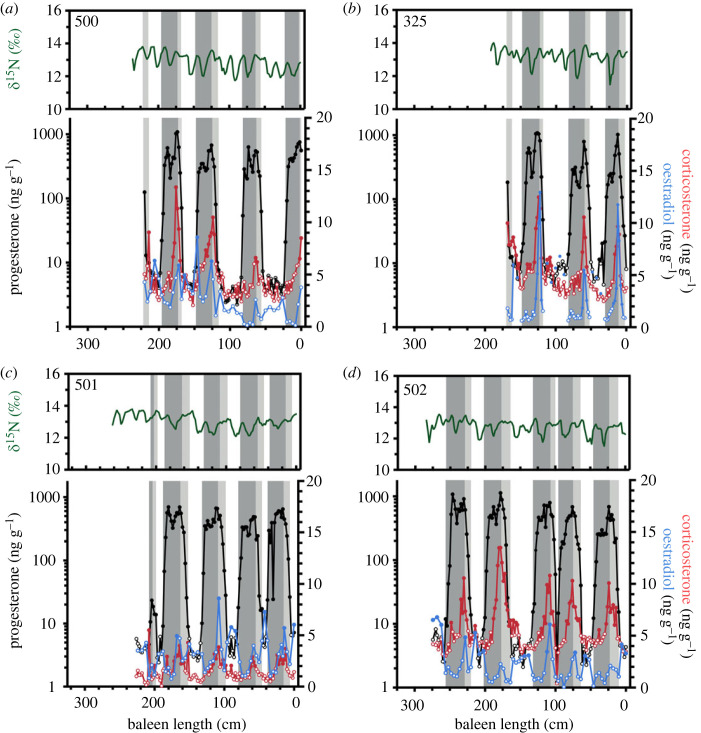
Profiles in stable nitrogen ratios (*δ*^15^N, green) and concentrations of immunoreactive progesterone (black), corticosterone (red) and oestradiol (blue) across the baleen plates of female bowhead whales hunted off west Greenland. Closed circles indicate hormone concentrations above a baseline threshold, while open circles indicate hormone concentrations within baseline levels. Dark grey box highlights gestation as defined under hypothesis 2; light grey box extends gestation as defined under hypothesis 1.

**Figure 4.**
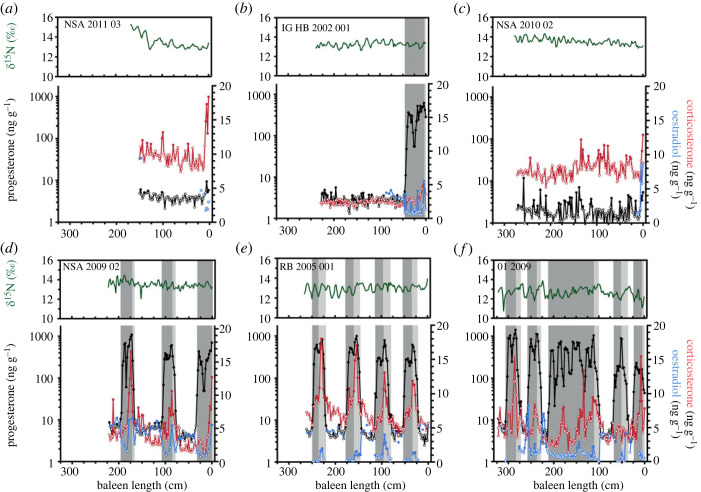
Profiles in stable nitrogen ratios (*δ*^15^N, green) and concentrations of immunoreactive progesterone (black), corticosterone (red) and oestradiol (blue) across the baleen plates of female bowhead whales hunted in the eastern Canadian Arctic. Closed circles indicate hormone concentrations above a baseline threshold, while open circles indicate hormone concentrations within baseline levels. Dark grey box highlights gestation as defined under hypothesis 2; light grey box extends gestation as defined under hypothesis 1.

The baleen of seven of the 10 females, ranging in length from 14.35–17.29 m, contained repeated, sustained elevations in P4 (figures 3 and 4). In general, P4 concentration increased from less than 10 to more than 100 ng g^−1^ during each elevation. Initial increases in baleen P4 tended to coincide with an increase towards seasonal maximum *δ*^15^N ratios, indicative of the late winter/early spring season (e.g. figure 2). Progesterone peaks were bimodal; with P4 being the only hormone with sustained elevations above baseline in the first phase of the peak. Corticosterone (B) peaks were observed in the second phase, and these persisted to the end of the P4 peak (figures 2–4). Oestradiol was frequently elevated during the second phase of bimodal P4 peaks (figures 2–4).

Under hypothesis 1 (gestation includes all P4 values above baseline), gestation of the seven adult female whales lasted a mean of 23.6 ± 1.5 months (range = 21.6–25.6 months; table 2). The mean duration of the first phase of the P4 peak was 10.0 ± 1.4 months (range = 8.0–12.0 months; table 2), and the mean duration of the second phase of the P4 peak was 13.6 ± 1.5 months (range = 11.6–16.0 months; table 2) The mean interpeak interval was 20.9 ± 11.1 months; with considerable intra- and inter-individual variability (range 12.1–43.3 months; table 2). Under hypothesis 2 (gestation ends at the last P4 maxima), mean gestation was 16.2 ± 1.5 months (range 14.3–19.2 months; table 2). The mean interpeak interval was 28.1 months, again with considerable intra- and inter-individual variability (range 19.1–49.2 months; table 2). The single shortest interpeak interval observed was 2.8 or 8.8 months (depending on how gestation was defined; individual 502) and the single longest was 48.3 or 53.3 months (4.0 or 4.4 years; individual NSA 2009-02) (figures 3 and 4). Given these observations, under either definition of gestation, the average calving interval (i.e. gestation + interpeak interval) for the seven whales was 3.7 years (range 2.9–5.7 years; table 2).

**Table 2.** Estimates of baleen stable nitrogen isotope (*δ*^15^N) period and mean duration of gestation, interpeak, and calving intervals of individual bowhead whales. (Reproductive cycle parameters were estimated under two hypotheses: (H1) inclusive of total progesterone (P4) peak and (H2) inclusive of P4 peak up to final maxima. Immature whales were not included in this analysis. nd, not determined.)

whale sample identity	*δ*^15^N period (cm yr^−1^)	H1: P4 total				H2: P4 max		calving interval (years)
		full P4 peak (months)	P4 1^st^ phase (months)	P4 2^nd^ phase (months)	interpeak interval (months)	gestation (months)	interpeak interval (months)	
NSA-2011-03	nd	nd	nd	nd	nd	nd	nd	nd
IG-HB-2002-001	20.2	nd	nd	nd	nd	nd	nd	nd
NSA-2010-02	26.3	nd	nd	nd	nd	nd	nd	nd
NSA-2009-02	14.4	25.0	10.8	14.2	43.3	19.2	49.2	5.7
RB-2005-001	15.0	25.6	12.0	13.6	26.7	16.8	35.2	4.3
01-2009	16.0	24.0	11.0	13.0	13.9	14.3	23.3	3.1
500	15.9	21.6	10.0	11.6	20.0	15.5	26.0	3.5
325	16.0	22.0	10.0	12.0	18.0	16.0	24.0	3.3
501	17.9	22.8	8.3	14.5	12.1	15.4	19.1	2.9
502	17.4	24.0	8.0	16.0	12.4	16.0	20.0	3.0
mean	16.1	23.6	10.0	13.6	20.9	16.2	28.1	3.7

The remaining three females, ranging from 9.04–14.32 m in total length, had uniformly low P4 concentrations across the majority of their baleen plates (figure 4). One of these females (IG-HB-2002-001, total length = 14.19 m) exhibited one bimodal P4 peak in its most recent growth (0–46 cm; figure 4*b*). Although probably indicative of this whale's first pregnancy, this P4 peak was incomplete since its baleen was excised at the gumline and excluded unerupted baleen and was therefore excluded from analyses of gestation events (see Methods). The other two females (NSA-2011-03 and NSA-2010-02) had sporadic elevations in P4 and corticosterone, but neither exhibited sustained P4 peaks (figure 4).

## 4. Discussion

**Figure 5.**
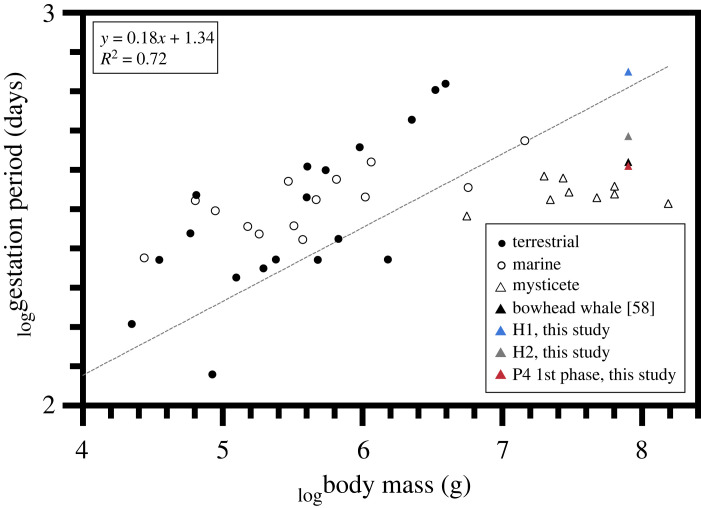
Regression of log-transformed mammalian body mass versus gestation period [60]. Symbols indicate the following: closed circles, terrestrial mammals; open circles, marine mammals; open triangles, other mysticete species; coloured triangles, bowhead whales (black, previous gestation estimate of 13.9 months [58]; blue, gestation estimated under hypothesis 1 (H1, this study); grey, gestation estimated under hypothesis 2 (H2, this study); red, second phase of bimodal P4 peak (this study)).

The prevailing paradigm of mammalian reproductive physiology indicates that P4 is elevated during pregnancy [45,46]. In free-ranging large whales, high P4 (i.e. several orders of magnitude higher than in individuals of other reproductive states) has been linked to gestation (reviewed in [11], but see [47]). Accordingly, pregnancy in several mysticete species has been assessed via P4 analysis of skin [48], blubber [5,49–51], faeces [52], respiratory vapour [53,54], and baleen [19–24]. Applying the conventional interpretation that high tissue P4 indicates pregnancy status (i.e. hypothesis 1), we would infer that bowhead whale gestation lasts 23.6 months—considerably longer than previous estimates of 13.9 months, based on observations of mating season, fetal growth and parturition dates, and Bayesian modelling [55–58]. The duration of progesterone peaks was determined using annual baleen growth rates—inferred from the periods of *δ*^15^N cycles derived from autoregressive models fitted across an entire baleen plate. This analysis could potentially obscure interannual variability in baleen growth related to ageing [59] or physiological changes, including pregnancy or lactation. However, we typically observed two annual *δ*^15^N cycles coincident with progesterone peaks (figures 2–4), confirming that baleen growth rate did not increase substantially over periods of elevated progesterone concentrations. Furthermore, bowhead whales, like other mysticetes, have shorter gestations than expected based on their body size, and a longer gestation better fits allometric predictions (figure 5; data from [60]). However, bowheads and other mysticetes experience seasonality thought to constrain their reproduction.

Field observations of bowhead mating behaviour (January–May) and parturition (late spring-early summer; [29]) could align with a gestation lasting approximately 2 years, but observed trends in fetal growth do not. Decades of observations following subsistence hunts reveal that bowhead whale fetuses are either small (fetal length < 100 cm) or large (greater than 350 cm) in the spring, and of intermediate size (approx. 200–300 cm) in the autumn [39,57,58]. Neonate calves, born in the late spring to early summer, are more than 400 cm in total length. If gestation was extended over nearly 2 years, we would expect to observe fetuses of intermediate length in the spring following the first year of gestation, as well as bimodal variation in the lengths of fetuses in the autumn (representing the first and second autumn after conception).

Given these observations, an approximately 2 year gestation is plausible with the occurrence of a reproductive delay (in fertilization or implantation), a period of polyestry, or both. Delayed fertilization may provide opportunities for post-copulatory sexual selection, including sperm competition [61,62] in polyandrous species such as balaenid whales where one female mates with many males in quick succession. Male balaenids, including bowhead and right whales, have larger than expected testes [63–66] and experience seasonal increases in testosterone production [67,68], suggesting the importance of sperm competition. Delayed implantation can allow females to match the energetic demands of pregnancy and lactation with favourable environmental conditions and optimal food abundance, or to selectively abort an embryo in suboptimal conditions [61,69]. Delayed implantation is common in some mammalian orders (e.g. pinnipeds, mustelids, ursids; reviewed in [61,70]), but has only been observed in one cetartiodactyl species, the roe deer (*Capreolus capreolus*; [71,72]). Delayed implantation is not thought to occur in cetaceans (reviewed in [2,73]), although gross inspections of whale carcasses could overlook small dormant blastocysts in pregnant females. Species exhibiting delayed implantation exhibit surges in P4 and/or oestradiol with implantation and the transition to active gestation [74,75], which is not inconsistent with findings of this study: the second phase of bimodal P4 peaks have a P4 surge and concurrent oestradiol elevations (figures 2–4).

An alternative explanation is that the baleen P4 peaks reflect a period of prolonged oestrus or sequential ovulations (seasonal polyestry, see [3]) preceding gestation. Progesterone elevations can occur in advance of implantation owing to the secretions of the corpus luteum (CL), a temporary endocrine structure formed from the ruptured ovarian follicle following ovulation. The CL secretes P4 to prepare the uterine lining for the implantation of a blastocyst [12], and continues to secrete P4 during early pregnancy prior to development of the placenta. High tissue P4 values have been associated with ovulation in cetaceans [50,53,76], with sequential ovulations and concurrent P4 elevation for 86 and 103 days, respectively, in captive odontocetes [77]. Mature bowhead whale ovaries can contain several corpus albicantia (CAs; formed from the involution of a CL) of similar size in the same ovary, signalling that several ovulations occurred close in time [29,78]. Bowhead whale mating behaviour has been observed from winter through to summer (M.P. Heide-Jørgensen 2018, unpublished data), and mating behaviour of north Atlantic right whales has been observed year-round [79–81]. A prolonged oestrous season in these species could allow females to maximize their reproductive success by being sexually receptive over longer time periods and capitalizing on multiple copulations and/or sperm competition to determine the highest quality father for their offspring.

The bimodal shape of the baleen P4 peaks, coupled with elevated corticosterone (B) and oestradiol during the second half (figures 2–4), point to a biphasic event consistent with either interpretation outlined above, in which the second phase of a P4 peak represents the active gestation phase. Glucocorticoids like corticosterone play an important role in fetal organ development during gestation [82,83]. Moreover, oestrogens (the most prominent being oestradiol) increase rapidly during the middle of pregnancy and remain elevated until parturition [84]. The second phase of the P4 peaks (with coincident corticosterone and sporadic oestradiol elevations) had a mean duration of 13.6 months (range = 11.6–16.0 months; table 2; figures 3 and 4), which aligns more closely to the field-derived estimate of an approximately 14 month gestation for bowhead whales. Previous work on north Atlantic right whales by Lysiak *et al*. [21] and Hunt *et al*. [22] similarly observed (i) baleen P4 peaks that are markedly longer (range = 15–23 months, *n* = 3 individuals) than gestation estimates derived from field observations and gross anatomy (12–13 months; [80,85]), and (ii) sustained elevations of corticosterone and oestradiol in the second phase of P4 peaks. Similar observations in two closely related species suggest certain unique aspects of balaenid whale endocrine profiles.

Alternatively, hypothesis 2 (in which we define gestation as a shorter component of the overall P4 peak) provides a mean gestation of 16.2 ± 1.5 months (range = 14.3–19.2 months; table 2), also closer to field-derived estimates of approximately 14 months [58]. This interpretation follows the logic that as pregnancy progresses, the placenta becomes a principal tissue of steroidogenesis [86,87]. Following expulsion of the placenta during parturition, circulating levels of P4 return to baseline levels [50]; but given their massive body size, the half-life of circulating hormone levels could be substantial in bowhead whales. Although more in alignment with current gestion estimates, this interpretation provides no explanation for the bimodal nature of the P4 peak, nor the concurrent increases in the other hormones in its latter half. Further work examining longitudinal endocrine profiles, analysing additional hormones, more individuals, and additional species is needed to better understand baleen whale reproductive physiology, including the ways in which they may diverge from the generalized mammalian endocrine model.

Competing interpretations of baleen hormone profiles with respect to gestation do not impact estimates of calving interval (only how it is partitioned between gestation and interpeak interval). The mean calving interval of 3.7 years (range 2.8–5.7 years) across the seven mature individuals is in close agreement with previous estimates for the Bering-Chukchi-Beaufort (BCB) bowhead population of 3–4 years [88], 3.06 years [89], or 3.15 years [38] determined using data from postmortem observations, ice-based censuses, and aerial surveys. However, we observed variation within and among individuals (table 2), which could reflect the influence of age/experience, body size, environmental conditions, and resource availability. Like all mysticetes, bowhead whales are capital breeders that rely on a thick blubber layer to support gestation and lactation [90–93], and previous studies have found that variable birth intervals in bowheads may cycle with climatic oscillations [56] or other indices of food availability.

We observed some deviations from the typical pattern of baleen P4 expression in two females and cite them as evidence of potential reproductive dysfunction or pregnancy loss. Female 01-2009 had a single, sustained P4 elevation for approximately 7 years (from baleen length 100–212 cm) with some coincident corticosterone elevations and sporadic increases in oestradiol (figure 4*f*). This region of baleen may indicate approximately 5 years of unsuccessful reproductive cycles or pregnancies, finally ending in an approximately 2 year gestation phase (as defined under both hypotheses explored in this paper) from baleen length 100–128 cm. Female 502 had the single shortest interpeak interval at 2.8 or 8.8 months (depending on how gestation was defined, from baleen length 98–106 cm; figure 3*d*). The interpeak interval includes lactation as well as a recovery period following weaning, where adult females replenish their lipid reserves and energy capital for subsequent calving events [90–93]. If peri- or neonatal mortality occurs, a female could conceivably be in positive energy balance and may be able to support a new pregnancy without a prolonged recovery period.

The uniformly low P4 concentrations of three females ranging from 9.04 to 14.32 m in length indicate these animals were immature. For comparison, examination of ovaries for CL or CA in the BCB bowhead whale population indicated that females less than 12.6–14.2 m were sexually immature [29,78]. Of these three females, IG-HB-2002-001 had baleen containing one P4 peak near the proximal (most recent) growth, which we interpret as her first pregnancy. Aspartic acid racemization of the eye lens from this animal estimated her age at 32 years and given that the unerupted baleen tissue from this animal was not sampled, we estimate that her age conception was approximately 28–29 years (table 1; figure 4). IG-HB-2002-001 had elevated oestradiol in the approximately 1.5 years preceding conception, indicating that she may have entered sexual maturity. We did not quantify oestradiol in older portions of this animal's baleen, so an accurate estimate of age at sexual maturity is not possible in this case, though our observations are in line with previous field studies estimates of bowhead whale sexual maturity at approximately the age of 26 [27,78,88]. Future work directly investigating endocrine markers of sexual maturity is needed for this species. The inferred immature status (or recently mature whale IG-HB-2002-001) of these whales is consistent with independent studies that reveal spatial segregation by sex, age and reproductive status among EC-WG bowhead whales. These three whales were among four collected from Foxe Basin and Gulf of Boothia, which are known aggregation areas for juvenile bowhead whales [94]. Moreover, upwards of 85% of animals that aggregate at Disko Bay, Greenland, are mature females [95], which is also consistent with the regular P4 peaks exhibited in baleen of each of the four animals sampled there.

## 5. Conclusion

This study reports on novel, retrospective, longitudinal baleen endocrine profiles from 10 female bowhead whales, providing, to our knowledge, the first population-specific calving interval estimates for EC-WG bowhead whales. The extended, bimodal P4 peaks in mature female baleen suggest that bowhead whale reproductive cycles are longer and more complex than previously assumed. We suggest these bimodal P4 peaks potentially reflect a biphasic event, with the first component corresponding to either a period of delayed implantation, development, or prolonged oestrus followed by active gestation, marked by concurrent elevation of all three hormones—progesterone, corticosterone, and oestradiol. Evolutionarily, both strategies have advantages which would allow females to maximize their reproductive success in Arctic environments with high interannual variability in environmental conditions. Alternatively, longitudinal P4 records of gestation may be more appropriately defined as ending prior to tissue hormone concentrations fully returning to baseline values. Future studies should examine additional hormones that could be included in the baleen endocrine profile (e.g. prolactin, relaxin or other progestagens), including higher temporal resolution analysis of oestrogens, to better assess evidence (or lack thereof) for delayed implantation or polyestry in this species. Our results warrant consideration for future endocrine studies of cetacean tissues, especially in point samples from matrices like skin, blubber, faeces or respiratory vapour—as high tissue P4 concentrations may not mean that a female mysticete is currently pregnant (e.g. if hypothesis 2 is correct; see [50,53,87]). Furthermore, the observed elevations in corticosterone coincident with gestation, an internal stressor, are important to consider in the broader context of marine mammal stress physiology, which often connects elevated glucocorticoids to external stressors only. Our integration of high-resolution longitudinal hormone data from baleen, along with consideration of published field and gross observations from the BCB and EC-WG bowhead populations, reveals novel aspects of bowhead reproductive physiology that appear to diverge from the generalized mammalian reproductive endocrinology model.

## Data Availability

Data have been provided in this submission as a table/electronic supplementary materials [96].
